# Clinical characteristics in surviving and nonsurviving older patients admitted to the ICU

**DOI:** 10.1186/cc14623

**Published:** 2015-03-16

**Authors:** E Pappa, H Pavlou, M Eforakopoulou

**Affiliations:** 1 KAT-EKA General Hospital Kifisia, Athens, Greece

## Introduction

In recent years the proportion of older people admitted to the ICU has increased. A variety of clinical and physiological factors are associated with outcome in these patients. The aim of this study is to determine the clinical characteristics associated with survival of ICU mechanically ventilated older patients.

## Methods

We retrospectively studied 74 patients, aged >65 years, admitted to the ICU who underwent mechanical ventilation. Standard demographic, clinical, and physiologic data were recorded. We examined the significant differences in clinical characteristics between survivors and nonsurvivors using the Student *t *and chi-square tests.

## Results

The mean age of patients studied (43 men and 31 women) was 79 ± 6.4 years. The type of admission was surgical 18%, trauma 26% and medical 57%. The ICU mortality of these patients was 57% and it was not associated with gender and cause of admission to ICU. Patients who survived had lower Charlson Comorbidity Index (*P *< 0.05) and shorter duration of mechanical ventilation (*P *< 0.01). The episodes of ventilation-associated pneumonia, sepsis and renal failure were less frequently in survivors (*P *< 0.05). Also, in addition serum iron and cholesterol levels were significantly lower in nonsurvivors (*P *< 0.01).

## Conclusion

The mortality of ICU older patients is high. VAP, sepsis and renal failure are frequent complications in nonsurvivors. Pre-existing comorbidities considerably affect mortality.
